# Beliefs and Intention to Organ Donation in Saudi Arabia: An Online Cross-Sectional Survey

**DOI:** 10.3390/healthcare11121716

**Published:** 2023-06-12

**Authors:** Sami Alobaidi

**Affiliations:** Department of Internal Medicine, University of Jeddah, Jeddah 21493, Saudi Arabia; salobaidi@uj.edu.sa

**Keywords:** beliefs, intention, organ donation, theory of planned behaviour, Saudi Arabia

## Abstract

Objective: Despite concerted and directed efforts to increase organ donation, the gap between the need for organs for transplantation and the lack of donors has been increasing globally. Studies have shown that donor rates in countries in the Middle East, such as Saudi Arabia, are particularly low despite a relatively advanced healthcare system and supportive government policies. There are multiple psychosocial, cultural, religious, and structural factors, that influence the increase in the organ donation rate, and some of them may be unique to a country such as Saudi Arabia. The theory of planned behaviour (TPB) is an important theory utilised to study how various types of attitudes, beliefs, and norms, influence organ donation intention and practice. In this study, we aimed to explore the normative, behavioural, and control beliefs, among residents of Saudi Arabia. Methods: The current study was a cross-sectional online survey performed from 6 June 2021 to 31 December 2021, using a questionnaire prepared in GoogleTM form among the residents of Saudi Arabia. The questionnaire asked demographic factors and questions to explore the normative, behavioural, and control beliefs, regarding organ donation. Results: This study received 1245 valid responses. Among the study participants, only 19.6% were willing to register as an Organ/Tissue donors. The intention for organ donation showed a statistically significant positive association with beliefs that organ donation is a good thing (123.51, df 4, *p* < 0.001), could save somebody’s life (81.38, df 4, *p* < 0.001), could have a positive impact on life after death (114, df 4, *p* < 0.001), and provision of better social support to family (of the deceased) can increase organ donation (68.43, df 4, *p* < 0.001). Those who expressed normative beliefs that their intention to donate organs strongly depend upon their family’s lack of objection to allowing the donation of organs at the time of death (190.76, df 4, *p* < 0.001), their knowledge about the organ transplantation process (179.35, df 4, *p* < 0.001), their knowledge about the viewpoint of their religion regarding organ donation (120.345, df 4, *p* < 0.001), and their knowledge about the registration facilities (241.64, df 4, *p* < 0.001), were more willing for donating their organs. Perception of worry about the likelihood of getting lesser care from doctors in an emergency if registered as an organ donor (OR = 4.25, 95% CI 1.57–11.51), beliefs that provision of better social support to family (of the deceased) can increase organ donation (OR = 10.49, 95% CI 1.56–70.43), and concern for the emotions of their family members while organ are being taken (OR = 4.37, CI 1.57–12.23), were the strongest predictors influencing a definite intention for organ donation. Conclusion: This study found that most of the components under normative and behavioural beliefs showed positive correlation with a definite intention for organ donation, whereas most of the components under control beliefs showed negative correlation with a definite intention for organ donation, among Saudi population. Based on the study results, there is a need to promote awareness regarding the organ donation process, especially the religious permissibility of organ donation, among general public in order to promote organ donation.

## 1. Introduction

Organ transplantation is an important and successful treatment strategy to save the lives of patients with end-stage organ diseases [1]. Due to the effectiveness of this treatment method in reducing morbidity and mortality, and its role in improving the quality of life of the recipients, organ donation and transplantation are increasing globally [2]. However, despite concerted and directed efforts to increase organ donation, the gap between the need for organs for transplantation and the lack of donors has been increasing globally [1,3]. In order to achieve self-sufficiency in organ transplantation as envisioned by The World Health Organization (WHO), each country needs to plan, organise, and supervise transplantation practices by utilising resources available locally [2].

The first organ transplantation (renal transplantation from a living donor) was conducted in the Kingdom of Saudi Arabia (KSA) in 1979. The Saudi Centre for OrganTransplantation (SCOT) was established in 1985 to supervise all organ transplantation programs at the national level, and deceased donor transplantation also started in the same year. By the year 2016, there were twelve kidneys, three livers, two pancreases, two lungs, and one cardiac transplant program throughout KSA [2]. However, with the ever-increasing demand for transplantation considering the higher prevalence (9892 per 100,000 affected) of chronic kidney diseases (CKD), organ shortage has become the major barrier to the expansion of organ transplantation programs in KSA [4]. Studies have shown that donor rates in countries in the Middle East, such as Saudi Arabia, are particularly low, despite a relatively advanced healthcare system and supportive government policies [5]. Around 1396 living donors (living donor rate: 41 per million population (PMP)) and 118 deceased organ donors (deceased donor rate: 3.5 PMP) were registered in KSA in 2022 [6]. Though both the living donor rate and the deceased donor rate are gradually increasing in KSA, the magnitude of the increase is significantly lower for deceased organ donation.

There are multiple psychosocial, cultural, religious, and structural factors, that influence the increase in the organ donation rate, and some of them may be unique to a country such as KSA [7]. It is prudent to understand the factors influencing the decision-making of organ donation in order to effectively respond to the challenge of the ever-increasing gap between the demand and availability of organs for transplant in KSA. Previous studies have utilised the theory of planned behaviour (TPB) in order to explore how various types of attitudes, beliefs, and norms, influence organ donation intention and practice [8]. According to the TPB model, an individual’s behaviour is a consequence of his or her behavioural intention, which is a consequence of three closely related constructs; attitude towards the behaviour, subjective norm, and perceived behavioural control. Various studies exploring the TPB model in organ donation found a linear positive correlation, with stronger attitudes, subjective norms, and PBC, leading to higher behavioural intention to donate organs and organ donation practice. A recent review regarding the application of the TPB model on organ donation behaviour demonstrated that TPB-based interventions carry the potential to improve the organ donation rate [9]. However, the moderating role of each TPB construct in influencing organ donation decisions can vary in different cultures. Studies exploring organ donation intention from multiple countries across the world utilising the TPB model showed varied findings. Some studies documented that all three constructs (attitude, subjective norm, and perceived behavioural control) significantly influence organ donation decisions in some countries, whereas another set of studies showed the superiority of one of the constructs over others in influencing organ donation decision-making [10,11,12]. Moreover, studies also reported culture-specific factors mediating the role of TPB constructs in influencing organ donation decision-making [12,13]. Religious views regarding organ donation can significantly influence the intention to organ donation among the population of KSA. Hence, considering the conflicting study findings and the significant role of cultural beliefs, it is important to explore the organ donation-decision making process and behaviour in every culture using the TPB model.

There are only a few studies exploring the organ donation decision-making process and behaviour in countries in the Middle East. A recent survey study among a sample of 1044 adults from Qatar using the TPB model found that behavioural and normative beliefs were significant positive predictors of the intention to donate organs, and control beliefs were found to be negatively associated with the organ donation intention [13]. To the best of our knowledge, there are no studies exploring the organ donation decision-making process and behaviour using a TPB model from KSA. To fill this gap, we conducted a large-scale online survey using a questionnaire based on the TPB model to explore the predictors of organ donation intention among the population of KSA. The aim of the present study was to explore the beliefs and intention toward organ donation in KSA and the influence of the TPB model on organ donation decision-making.

## 2. Methodology

### 2.1. Study Design and Participants

This study was an online cross sectional survey study using a questionnaire prepared in GoogleTM form conducted among the residents of KSA from 6 June 2021 to 31 December 2021. The study protocol was approved by the institutional ethical committee with approval code UJ-REC-020.

The questionnaire was adapted from a study investigating the beliefs and intention for organ donation in Qatar [13]. The purpose of the study was provided in the initial part of the questionnaire and participants could progress to the study questionnaire only after explicit agreement about participation in the study.

#### 2.1.1. Instruments

The study questionnaire consisted of three sections. The first part of the study questionnaire asked about the socio-demographic variables such as age, gender, nationality, religion, marital status, education, occupation, average monthly income, and location. The second part of the study questionnaire enquired about general awareness of organ donation. The last part of the study questionnaire was based on the TPB model. In order to investigate the study participants’ normative, behavioural, and control beliefs regarding organ donation, TPB constructs were added to the study questionnaire. A five-point scale (strongly agree, agree, neutral, disagree, and strongly disagree) was used to rate participants’ agreement with statements.

#### 2.1.2. Statistical Analysis

The Statistical Package for Social Sciences (SPSS Inc., Chicago, IL, USA, version 26.0 for Windows) was used to analyse the data. Descriptive statistics were utilised to analyse socio-demographic variables. The association between intention to organ donation and beliefs was tested using chi-square tests. The predictors of definite intention to organ donation were analysed using binary logistic regression, where the intention to organ donation (1—definite intention to organ donation, 0—does not intend to organ donation) was defined as the dependent variable. In order to calculate odds ratios (OR) and 95% confidence intervals (CI), the forward LR method of regression was utilised. The level of statistical significance was maintained at *p* < 0.05.

## 3. Results

Sociodemographic details were summarised in Table 1.

## 4. Behavioural Beliefs

The majority of the study participants (79.7%) agreed that organ donation could have a positive impact on life after death and they will be rewarded by God for such an act. Around 79.4% of the study participants agreed that organ donation is a good thing and should be promoted, and 91.6% agreed that organ donation could save somebody’s life. Only 24.9% of the study participants agreed with the statement that patients registered for organ donation receive lesser care from doctors in case of an emergency, and only 28.6% agreed that the organ retrieval process after death may cause body disfigurement. Finally, 68.2% agreed that provision of better social support to families (of the deceased), regardless of whether they donate or not, can increase organ donation.

### 4.1. Normative Beliefs

Around 51.7% of the study population expressed their wish to take the opinion of others before registering as an organ donor in Saudi Arabia, mainly from family members. A total of 61.4% agreed that they would be more willing to register as organ donors provided their family members have no objection. A total of 68.6% and 70.1% agreed that their willingness for organ donation would be more if they have more knowledge about organ donation and if they know more about the viewpoint of their religion regarding organ donation, respectively.

### 4.2. Control Beliefs

Among the study respondents, only 11.6% agreed that they may find many opportunities to register as organ donors in KSA, and only 22.8% agreed that they may get answers to all their questions while registering. A total of 44.3% of the study participants agreed that they are healthy to donate organs. Only 24% agreed that operation procedure related to organ donation is encouraging, and 21.9% disagreed with the statement that organ donation might leave you weak and disabled. More than half of the study participants (64.1%) expressed their trust in the healthcare system in KSA. Around 56.7% also agreed that they are concerned with the emotions of their family members related to organ donation.

### 4.3. Intentions to Donate Organs

Among the study participants, only 19.6% were willing to register as an Organ/Tissue donor in KSA. A total of 26.6% agreed that they will consider organ donation after a discussion with a religious leader, and 43.9% agreed that they will consider donating an organ more seriously if they are approached by a trusted organization.

The intention for organ donation showed a statistically significant positive association with behavioural beliefs that organ donation is a good thing (123.51, df 4, *p* < 0.001), could save somebody’s life (81.38, df 4, *p* < 0.001), could have a positive impact on life after death (114, df 4, *p* < 0.001), and provision of better social support to family (of the deceased) can increase organ donation (68.43, df 4, *p* < 0.001). Moreover, the belief that patients registered for organ donation receive lesser care from doctors in case of an emergency (30.06, df 4, *p* < 0.001), organ retrieval process after death may cause body disfigurement (15.88, df 4, *p* = 0.003), showed a statistically significant negative association with a definitive intention for organ donation.

The chi squire test also showed that those who expressed normative beliefs that their intention to donate an organ strongly depend upon their family’s no objection for allowing the donation of an organ at the time of death (190.76, df 4, *p* < 0.001), their knowledge about organ transplantation process (179.35, df 4, *p* < 0.001), their knowledge about the viewpoint of their religion regarding organ donation (120.345, df 4, *p* < 0.001), and their knowledge about the registration facilities (241.64, df 4, *p* < 0.001), were more willing for donating their organs, Those who expressed the need for opinion of others before registering as an organ donor (11.66, df 1, *p* = 0.001) were less likely to donate their organs.

The chi squire test also showed that those expressed control beliefs that they were healthy to donate (29.02, df 4, *p* < 0.001), have no age limitation for donating their organs (30.87, df 4, *p* < 0.001), and organ registration as a time-consuming process (10.04, df 4, *p* = 0.040), where more willing for donating their organs. On the other hand, those who expressed control the beliefs that operation procedure for procuring organs was discouraging (31.26, df 4, *p* < 0.001), organ donation might leave them weak and disabled (36.22, df 4, *p* < 0.001), and concern for the emotions of their family members while organ are being taken (51.86, df 5, *p* < 0.001), were less likely to donate their organs.

In logistic regression analysis, perception of worry about the likelihood of getting lesser care from doctors in an emergency if registered as an organ donor (OR = 4.25, 95% CI 1.57–11.51), beliefs that provision of better social support to family (of the deceased) can increase organ donation (OR = 10.49, 95% CI 1.56–70.43), and concern for the emotions of their family members while the organ is being taken (OR = 4.37, 95% CI 1.57–12.23), were the strongest predictors influencing a definite intention for organ donation.

The association between intention to organ donation and beliefs were summarised in Table 2.

## 5. Discussion

The present study evaluated the intention towards donating organs among the population of KSA using the TPB model, and found that normative, behavioural, and control beliefs significantly influence organ donation decision-making among the Saudi population. Similar results were also reported in a recent study from Qatar where investigators explored beliefs and intentions to organ donation using the TPB model among 1044 adults utilising a household survey design [13]. Another study evaluating organ donation intentions in Americans and Koreans also highlighted the significant influence of TPB components while taking organ donation decisions [14]. The current study also found that most of the components under normative and behavioural beliefs showed a statistically significant positive correlation with a definite intention for organ donation, whereas most of the components under control beliefs showed a statistically significant negative correlation with a definite intention for organ donation, which is consistent with previous study results. The logistic regression results also failed to show any component of control beliefs predicting organ donation intention. The overall results indicate that normative and behavioural beliefs significantly influence organ donation intention among the Saudi population, and control beliefs had a relatively less significant role of control beliefs in predicting organ donation, which is consistent with the existing literature [8,13].

Globally policies for collecting organs for transplantation and advocacy systems for encouraging organ donation were founded on the paradigm of altruistic behaviour [15]. Organ donation is an ultimate example of an altruistic act where altruistic individuals create a norm of volunteering for organ donation leading to social solidarity [16]. In this study, around 79.4% and 91.6% of the participants agreed with the altruistic beliefs that organ donation is a good thing and should be promoted, and organ donation could save somebody’s life, respectively. Moreover, this study also found a statistically significant positive correlation between altruistic beliefs and definite intention for organ donation. Similar results were also found in a recent study from Qatar where 95% of individuals agreed with altruistic beliefs toward organ donation [13]. This study’s results indicates that the Saudi population is generally altruistic with respect to organ donation, and policies aiming at creating opportunities for individuals for altruism in the form of registering for organ donation may promote social solidarity.

One of the major issues facing organ donation projects in the middle eastern countries is the lack of growth in the rate of deceased organ donation when compared to voluntary organ donation [2]. Studies had shown that providing support to grieving families during a painful time of loss could promote deceased organ donation [17]. Around 68.2% of the study participants also agreed with this idea that the provision of better social support to family (of the deceased), regardless of whether they donate or not, can increase organ donation. A similar study from Qatar also reported that 63.7% of the study participants agreed with the above idea [13]. Based on the existing literature and the current study findings, it is prudent to create opportunities to support organ donors and their families in order to promote organ donation. Recently, the King Abdulaziz Medal of Third Class was awarded to 200 Saudi citizens by King Salman as appreciation for their registration in a government organ donation program [18]. Moreover, the Tawakkalna application, in cooperation with the Saudi Center for Organ Transplantation (SCOT), also awarded three types of medals—gold, silver and bronze—to organ donors, in appreciation of their sacrifices for patients suffering from organ failures [19]. All these recent developments indicate that the government of KSA is aware of the importance of providing support to donors. However, there is a need to create organ procurement organizations, similar to the Gift of Life Donor Program (GLDP) in Philadelphia, to work closely with grieving families in order to support them and encourage them toward deceased organ donation [17].

The influence of Islamic religious beliefs on organ donation decision-making in KSA is not encouraging based on previously published literature. A cross-sectional study among a random sample of 948 Saudi citizens between 20–60 years of age showed that 27.5% feared that the act of organ donation contradicted their religious beliefs [20]. In another recent study, around 39.29% of the study participants who opposed organ donations suggested religious beliefs as the reason for opposing organ donations [21]. Even though, the Islamic Jurisprudence Assembly Council in Saudi Arabia approved deceased and live donation in a landmark decision in 1988, the overall data indicates a lack of awareness of the permissibility of organ donation in the religion of Islam in the Saudi population [22]. Similar to past studies, around 20.3% of the study participants in this study disagreed with the statement that organ donation could have a positive impact on life after death and they will be rewarded by God for such an act. Around 70.1% agreed that their willingness for organ donation would increase if they know more about the viewpoint of their religion regarding organ donation, and around 26.6% agreed that they will consider organ donation after discussion with a religious leader. The study results showed the significant impact of religious beliefs in decision-making for organ donation, as well as the role of religious leaders in influencing beliefs and intention to donate organs. There is a need to increase awareness about Islamic religious viewpoint regarding organ donation among the Saudi population, preferably by involving religious figures in awareness campaigns.

This study also found that some of the behavioural, normative, and control beliefs also showed a significant negative correlation with a definite intention to organ donation. There is a need to target such beliefs such as the perception of lesser medical care in emergencies after registering for organ donation, organ donation cause body disfigurement and might leave them weak and disabled, effectively through awareness campaigns and educational programs in order to improve the rate of organ donation in Saudi population.

The study results should be read keeping in mind a few limitations. The design of this study was an online survey in which responses were collected after distributing questionnaires on social media platforms. Such study designs often carry the risk of selection bias. Moreover, the characteristics of the study population and the overrepresentation of the western region limit the generalisability of the study results.

## 6. Conclusions

This study found that most of the components under normative and behavioural beliefs showed a positive correlation with a definite intention for organ donation, whereas most of the components under control beliefs showed a negative correlation with a definite intention for organ donation, among the Saudi population. Based on the study results, there is a need to promote awareness regarding the organ donation process, especially the religious permissibility of organ donation, among the general public in order to promote organ donation.

## Figures and Tables

**Table 1:** Sociodemographic and general enquiry description on organ donation in Saudi Arabia.

Variable		N (%)
Gender		
	Female	673 (54.1%)
	Male	572 (45.9%)
Nationality		
	Saudi	1117 (89.7%)
	Non-Saudi	128 (10.3%)
Religion		
	Muslim	1220 (98%)
	Christian	9 (0.7%)
	Others	16 (1.3%)
Marital Status		
	Married	622 (50%)
	Single	575 (46.2%)
	Widowed	48 (3.9%)
Education		
	High school and below	158 (12.7%)
	Diploma and Bachelor	820 (65.9%)
	Masters or PhD	267 (21.4%)
Monthly income		
	<5000	496 (39.9%)
	5001–10,000	231 (18.6%)
	10,001–15,000	210 (16.9%)
	>15,000	307 (24.7%)
Location		
	Central	163 (13.1%)
	Western	845 (67.9%)
	Eastern	125 (10%)
	Northern	50 (4%)
	Southern	62 (5%)
Heard of organ donation		
	No	17 (1.4%)
	Yes	1228 (98.6%)
Attended organ donation campaign (N = 1228)		
	No	1125 (91.6%)
	Yes	103 (8.4%)
Have donated blood		
	No	759 (61%)
	Yes	486 (39%)
Have donated organ/tissue		
	No	1230 (98.8%)
	Yes	15 (1.2%)

**Table 2:** Association between TPB variables and intention to organ donation.

Variable		Total Responses	Organ Donation Intention		*p* Value
			Definitely Yes	No/Not Decided	
Behavioural Beliefs					
Organ donation is a good thing					
	Strongly agree	416 (45.1%)	146 (80.7%)	270 (36.4%)	<0.001
	Agree	263 (28.5%)	32 (17.7%)	231 (31.2%)	
	Neutral	211 (22.9%)	3 (1.7%)	208 (28.1%)	
	Disagree	18 (2%)	0	18 (2.4%)	
	Strongly disagree	14 (1.5%)	0	14 (1.9%)	
Organ donation could save somebody’s life					
	Strongly agree	553 (60%)	161 (89%)	392 (52.9%)	<0.001
	Agree	267 (29%)	20 (11%)	247 (33.3%)	
	Neutral	86 (9.3%)	0	86 (11.6%)	
	Disagree	8 (0.9%)	0	8 (1.1%)	
	Strongly disagree	8 (0.9%)	0	8 (1.1%)	
Organ donation could have a positive impact on life after death					
	Strongly agree	435 (47.2%)	149 (82.3%)	286 (38.6%)	<0.001
	Agree	241 (26.1%)	22 (12.2%)	219 (29.6%)	
	Neutral	196 (21.3%)	8 (4.4%)	188 (25.4%)	
	Disagree	27 (2.9%)	2 (1.1%)	25 (3.4%)	
	Strongly disagree	23 (2.5%)	0	23 (3.1%)	
Provision of better social support to the family (of the deceased) can increase organ donation					
	Strongly agree	241 (26.1%)	89 (49.2%)	152 (20.5%)	<0.001
	Agree	347 (37.6%)	58 (32%)	289 (39%)	
	Neutral	270 (29.3%)	28 (15.5%)	242 (32.7%)	
	Disagree	34 (3.7%)	1 (0.6%)	33 (4.5%)	
	Strongly disagree	30 (3.3%)	5 (2.8%)	25 (3.4%)	
Patients registered for organ donation receive lesser care from doctors in case of an emergency					
	Strongly agree	101 (11%)	37 (20.4%)	64 (8.6%)	<0.001
	Agree	136 (14.8%)	17 (9.4%)	119 (16.1%)	
	Neutral	342 (37.1%)	50 (27.6%)	292 (39.4%)	
	Disagree	169 (18.3%)	38 (21%)	131 (17.7%)	
	Strongly disagree	174 (18.9%)	39 (21.5%)	135 (18.2%)	
Organ retrieval process after death may cause body disfigurement					
	Strongly agree	80 (8.7%)	12 (6.6%)	68 (9.2%)	0.003
	Agree	197 (21.4%)	29 (16%)	168 (22.7%)	
	Neutral	370 (40.1%)	67 (37%)	303 (40.9%)	
	Disagree	182 (19.7%)	43 (23.8%)	139 (18.8%)	
	Strongly disagree	93 (10.1%)	30 (16.6%)	63 (10.1%)	
Normative Beliefs					
I would be more willing to register as an organ donor if					
I know that my family would have no objection to allowing donation of organ at the time of death					
	Strongly agree	239 (25.9%)	115 (63.5%)	124 (16.7%)	<0.001
	Agree	243 (26.4%)	48 (26.55)	195 (26.3%)	
	Neutral	273 (29.6%)	14 (7.7%)	259 (35.0%)	
	Disagree	87 (9.4%)	2 (1.1%)	85 (9.4%)	
	Strongly disagree	80 (8.7%)	2 (1.1%)	78 (10.5%)	
I know more about organ transplantation process					
	Strongly agree	246 (26.7%)	116 (64.1%)	130 (17.5%)	<0.001
	Agree	315 (34.2%)	52 (28.7%)	263 (35.5%)	
	Neutral	236 (25.6%)	11 (6.1%)	225 (30.4%)	
	Disagree	73 (7.9%)	1 (0.6%)	72 (9.7%)	
	Strongly disagree	52 (5.6%)	1 (0.6%)	51 (6.9%)	
I know more about the viewpoint of their religion regarding organ donation					
	Strongly agree	310 (33.6%)	120 (66.3%)	190 (25.6%)	<0.001
	Agree	285 (30.9%)	45 (24.9%)	240 (32.4%)	
	Neutral	229 (24.8%)	13 (7.2%)	216 (29.1%)	
	Disagree	58 (6.3%)	0 (0.0%)	58 (7.8%)	
	Strongly disagree	40 (4.3%)	3 (1.7%)	37 (5.0%)	
I know how to I could register					
	Strongly agree	227 (24.6%)	121 (66.9%)	106 (14.3%)	<0.001
	Agree	255 (27.7%)	47 (26.0%)	208 (28.1%)	
	Neutral	297 (32.2%)	12 (6.6%)	285 (38.5%)	
	Disagree	84 (9.1%)	1 (0.6%)	83 (11.2%)	
	Strongly disagree	59 (6.4%)	0 (0.0%)	59 (8.0%)	
Control Beliefs					
They are not healthy to donate					
	Strongly agree	78 (8.5%)	9 (5.0%)	69 (9.3%)	<0.001
	Agree	187 (20.3%)	32 (17.7%)	155 (20.9%)	
	Neutral	305 (33.1%)	42 (23.2%)	263 (35.5%)	
	Disagree	235 (25.5%)	59 (32.6%)	176 (23.8%)	
	Strongly disagree	117 (12.7%)	39 (21.5%)	78 (10.5%)	
Your age is not fit for donating your organ					
	Strongly agree	41 (4.4%)	6 (3.3%)	35 (4.7%)	<0.001
	Agree	97 (10.5%)	15 (8.3%)	82 (11.1%)	
	Neutral	297 (32.2%)	38 (21.0%)	259 (35.0%)	
	Disagree	323 (35.0%)	67 (37.0%)	256 (34.5%)	
	Strongly disagree	164 (17.8%)	55 (30.4%)	109 (14.7%)	
Organ donor registration can be a time-consuming process					
	Strongly agree	34 (3.7%)	12 (6.6%)	22 (3.0%)	0.040
	Agree	126 (13.7%)	31 (17.1%)	95 (12.8%)	
	Neutral	418 (45.3%)	69 (38.1%)	349 (47.1%)	
	Disagree	231 (25.1%)	45 (24.9%)	186 (25.1%)	
	Strongly disagree	113 (12.3%)	24 (13.3%)	89 (12.0%)	
Operation procedure for procuring organs is discouraging					
	Strongly agree	164 (17.8%)	22 (12.2%)	142 (19.2%)	<0.001
	Agree	300 (32.5%)	58 (32.0%)	242 (32.7%)	
	Neutral	284 (30.8%)	44 (24.3%)	240 (32.4%)	
	Disagree	112 (12.1%)	31 (17.1%)	81 (10.9%)	
	Strongly disagree	62 (6.7%)	26 (14.4%)	36 (4.9%)	
You are worried that organ donation might leave you weak and disabled					
	Strongly agree	191 (20.7%)	29 (16.0%)	162 (21.9%)	<0.001
	Agree	358 (38.8%)	63 (34.8%)	295 (39.8%)	
	Neutral	211 (22.9%)	30 (16.6%)	181 (24.4%)	
	Disagree	106 (11.5%)	39 (29.5%)	67 (9.0%)	
	Strongly disagree	56 (6.1%)	20 (11.0%)	36 (4.9%)	
Emotions of your family members while organs are being taken make you feel concerned					
	Strongly agree	238 (25.8%)	27 (14.9%)	211 (28.5%)	<0.001
	Agree	321 (34.8%)	61 (33.7%)	260 (35.1%)	
	Neutral	196 (21.3%)	29 (16.0%)	167 (22.5%)	
	Disagree	91 (9.9%)	32 (17.7%)	59 (8.0%)	
	Strongly disagree	65 (7.0%)	28 (15.5%)	37 (5.0%)	

## Data Availability

Data is available on request.
